# *Chiliadenus iphionoides* Reduces Body Weight and Improves Parameters Related to Hepatic Lipid and Glucose Metabolism in a High-Fat-Diet-Induced Mice Model of NAFLD

**DOI:** 10.3390/nu14214552

**Published:** 2022-10-28

**Authors:** Gil Zandani, Sarit Anavi-Cohen, Tamar Yudelevich, Abraham Nyska, Nativ Dudai, Zecharia Madar, Jonathan Gorelick

**Affiliations:** 1 The Faculty of Agriculture, Food and Environment, The Hebrew University of Jerusalem, Rehovot 7670110, Israel; 2 School of Nutritional Sciences, Peres Academic Center, Rehovot 7610202, Israel; 3 Sackler School of Medicine, Tel Aviv University, Tel Aviv 6997801, Israel; 4 Unit of Medicinal and Aromatic Plants, Newe Ya’ar Research Center, Agricultural Research Organization, Ramat Yishay 30095, Israel; 5 Eastern Regional R&D Center, Kiryat Arba 90100, Israel

**Keywords:** NAFLD, Chiliadenus iphionoides, lipid metabolism, glucose tolerance

## Abstract

Non-alcoholic fatty liver disease (NAFLD) has become an epidemic with increasing prevalence. Limited treatment options and poor adherence emphasize the urgent need for novel therapies for the treatment and/or prevention of NAFLD. Bioactive natural compounds found in medicinal plants are promising as novel therapeutic agents for NAFLD. *Chiliadenus iphionoides,* a medicinal plant with several health-promoting properties, is an encouraging candidate. The current study aimed to elucidate the metabolic effects of *C. iphionoides* consumption in a high-fat-diet (HFD)-induced model of NAFLD. Male C57BL/6J mice (*n* = 40, 7–8-week-old) were fed a HFD (60% fat) with/without 0.5 or 2.5 gr *C. iphionoides* for fifteen weeks. Diet supplementation with *C. iphionoides* significantly ameliorated HFD-induced weight gain. Likewise, liver and adipose tissue weights were profoundly lower in the *C. iphionoides*-fed groups. Reduced liver steatosis in those groups was corroborated by histology, plasma liver enzyme levels, and lipid profile, indicating improved liver function and lipid metabolism in addition to enhanced insulin sensitivity. The addition of *C. iphionoides* to an obesogeneic diet can beneficially alleviate metabolic alterations and may be a practicable strategy for the management of NAFLD.

## 1. Introduction

Non-alcoholic fatty liver disease (NAFLD), or more recently termed Metabolic Dysfunction Associated Fatty Liver Disease (MAFLD), defined by macrovesicular steatosis in ≥5% hepatocytes in the absence of alcohol or drug use [1], is considered among the most common chronic liver diseases globally, with the numbers of affected individuals continuously rising. It is currently estimated that about a quarter of the general population suffers to some degree from above normal fat accumulation within liver tissue [2]. Liver steatosis occurs at even higher rates within the context of underlying conditions, i.e., obesity, type 2 diabetes, metabolic syndrome, and insulin resistance [3,4].

Despite being widespread, pharmacologic treatments of NAFLD are not well established. Current clinical data do not indicate significant improvement with several tested pharmacological therapies, probably due to the complexity of the disease and its high individual variability. With no current approved pharmacological treatments available, up-to-date recommendations involve predominantly lifestyle modifications focused on diet modifications and the promotion of an active lifestyle. However, while taking a pill is easy, changing one’s attitude toward food and physical activity is more difficult to implement. Thus, these options usually encounter barriers of low compliance, especially over long-term periods [5].

A medicinal plant is defined as “a plant comprising active ingredients or secondary metabolites that possess biological activity” [6]. Previous reports have emphasized the therapeutic potential of plants in controlling chronic diseases, suggesting attention should be given to complementary therapies that harness the use of traditional plants/functional foods as a novel treatment strategy for managing NAFLD, with a number plants such as flaxseed, cinnamon, and green tea showing promise [7]. The mixture of multiple bioactive food compounds working in synergy may be an efficacious approach for preventing and/or treating NAFLD, but there is still no plant-based treatment clinically proven to alleviate the disease. The search is still on to identify plants as potentially effective alternative treatments. 

The Chiliadenus genus, comprised of ten species, are predominantly distributed along the southern edge of the Mediterranean Sea [8]. One of these species, *Chiliadenus iphionoides* (Boiss.& Blanche) Brullo (Asteraceae), also known as *Varthemia iphionoides*, is traditionally used for pain relief, wound healing, treatment for eye complaints, and urine retention [9]. Depending on the indication, fresh leaves are applied externally, or taken internally as a water-based extract. *C. iphionoides* has been reported to possess various therapeutic properties, including anticancer, antimicrobial, antioxidant, antispasmodic, and antiplatelet activities [10]. The antidiabetic properties attributed to *C. iphionoides* are supported by in vitro and in vivo studies which documented reduced blood glucose, reduced intestinal glucose absorption, and enhanced insulin secretion capacity of pancreatic β cells [11,12].

The beneficial effects of *C. iphionoides* on NAFLD have not been evaluated. Therefore, the present work aimed to elucidate the effects of *C. iphionoides* supplementation to a high fat diet in mice on metabolic parameters related to NAFLD. 

## 2. Materials and Methods

### 2.1. Chiliadenus Iphionoides Plant Extract Preparation

Samples of *C. iphionoides* (Boiss. & Blanche) Brullo were collected from the Hebron Hills region near Moshav Carmel in Israel. A voucher specimen (# 10 25 32) was deposited at the Herbarium at the National Natural History Collection of the Hebrew University of Jerusalem (HUJ). Plants were air-dried and the aerial parts ground into a powder. One-hundred grams of powder was extracted in 1 L of 95% ethanol and allowed to stand at room temperature for 24 h. After filtration, the solution was concentrated under vacuum to a final concentration of 100 mg/mL. Next, 0.5 gr (5 mL) or 2.5 gr (25 mL) *C. iphionoides* was diluted to a final volume of 50 mL ethanol and added to experimental diets until a homogenous mixture was prepared. Comparable amounts of ethanol were added to the control diets. All experimental diets were dried to evaporate ethanol before testing. The tested concentrations were based on our initial cellular assays, as well as previous animal experiments [11].

### 2.2. Experimental Animals and Diets

Male C57BL/6J mice, 7–8 weeks old, were purchased from Harlan Laboratories (Jerusalem, Israel). All experimental protocols used in the animal experiments were performed according to the guidelines of the Authority for Biological and Biomedical Models and were approved by the Institutional Animal Care Ethics Committee of the Hebrew University of Jerusalem (AG-18-15708-3). Mice were housed under a controlled atmospheric environment (12/12 h light/dark cycle, 18–24 °C, humidity 60%) and provided with food and water at all times. Following the acclimatization period, the mice (*n* = 40) were randomly divided into four groups (*n* = 10 per group): (1) normal diet (ND), (2) high-fat (60%) diet (HFD), (3) high-fat (60%) diet + 0.5 gr *C. iphionoides* ~100 mg/kg (HFD + 0.5CI), and (4) high-fat (60%) diet + 2.5 gr *C. iphionoides* ~500 mg/kg (HFD + 2.5CI) for fifteen weeks. The diet compositions are presented in Table 1. All mice were allowed free access to food and water throughout the experiment. Their body weight (BW) and food intake were recorded weekly. 

### 2.3. Oral Glucose Tolerance Test (OGTT)

Glucose-loading tests were conducted in week 13 of the experimental period. The mice were fasted overnight before the OGTT, then weighed and marked. At time 0, an initial baseline glucose measurement was taken. The mice were then given D-glucose (3 g/kg body weight) via gavage. Glucose levels in blood samples were obtained from tail veins and measured at 30, 60, and 120 min after glucose loading with a glucometer (handheld Optimum Xceed Glucometer, Abbott Diagnostic Care Ltd., Berkshire, UK.).

### 2.4. Homa-IR

The insulin resistance index was estimated by the homeostasis model assessment (HOMA) parameter using the following equation: HOMA = fasting serum insulin (μU/mL) × fasting plasma glucose (mM)/22.5 [13].

### 2.5. Animal Sacrifice and Organ Collection

At the end of the experiment, the mice were fasted overnight, their BW was recorded, and they were sacrificed in random order by isoflurane (Minard Inc., USA) anesthesia. Blood was collected from the vena cava, centrifuged at 8000 rpm at 4 °C for 10 min, and stored at −80 °C. Adipose tissue was removed, weighed, placed in liquid nitrogen, and stored at −80 °C. Liver tissue was collected and weighed. A small sample from the right liver lobe was placed in 4% formaldehyde, and the remaining liver tissue was minced in liquid nitrogen and stored at −80 °C.

### 2.6. Biochemical Analysis of Serum Parameters

Liver enzymes: serum alanine aminotransferase (ALT) and serum aspartate aminotransferase (AST) were measured with an automated clinical chemistry analyzer along with total cholesterol, high-density lipoprotein (HDL), and total triglycerides (American Laboratories Ltd., Herzliya, Israel). Concentrations of plasma insulin were determined by a Rat/Mouse Insulin ELISA Kit (Cat #EZRMI-13K), supplied by Merck (Rehovot, Israel).

Automated quantification of fatty vacuoles in the liver was performed using artificial intelligence (AI).

In each liver section, five different microscopic fields (magnification × 600) were selected by a board-certified pathologist, all located in the centrilobular regions. These microscopic fields were photographed with the microscope-based digital pathology system and analyzed in real-time using the AI application for automated quantification of fatty vacuoles in the liver. The AI application (AIRA Matrix, Mumbai, India) used in this study is based on a semi-automated algorithm [14].

### 2.7. Liver and Intestine Histology Examination

Histological slides were prepared by Patholab (Rehovot, Israel). Livers and colons were macro-dissected, placed in plastic cassettes, and dehydrated. The dehydrated samples were embedded in paraffin blocks by an automatic apparatus. Serial sections 3–5 μm thick were cut from each block, placed on glass slides, stained with hematoxylin and eosin (H&E), and covered by an automatic apparatus. The histopathological examinations were performed by Dr. Abraham Nyska, DVM, Dipl. ECVP, Fellow IATP, board certified in toxicologic pathology—https://ebvs.eu/colleges/ECVP/members/prof-abraham-nyska (accessed on 7 November 2021). Histopathological changes were described and scored by the study pathologist, using semi-quantitative grading of five grades (0–4), taking into consideration the severity of the changes. The scoring reflects the predominant degree of the specific lesion seen in the entire field of the histology section. A generic grading criterion was used [15]: Zero (0) = no lesion; 1 = minimal change; 2 = mild change; 3 = moderate change; and 4 = marked change.

### 2.8. Western Blot Analysis

Liver tissues lysates were prepared using lysis buffer as previously described [16]. Blots were respectively incubated with dilutions of primary antibodies (AMPK 1:1000, #2532 Cell Signaling Technology; p-AMPK 1:1000, #2531 Cell Signaling Technology; ACC 1:1000, #3662 Cell Signaling Technology; p-ACC 1:1000 #3661 Cell Signaling Technology; AKT 1:1000 #9272 Cell Signaling Technology; p-AKT 1:1000, #9271 Cell Signaling Technology; β actin 1:1000, #3700 Cell Signaling Technology) at 4° overnight. After several washes, the membranes were incubated with a secondary goat antibody at 1:10,000 dilution (Jackson Immuno-Research Laboratories, West Grove, PA, USA). The immune reaction was detected by enhanced chemiluminescence (Gel ChemiDoc MP System (Bio-Rad, Hercules, CA, USA)), with bands being quantified by densitometry and expressed as arbitrary units. The band optical density was analyzed on an Image lab system (Bio-Rad, Hercules, CA, USA), and β-actin was used as a control protein.

### 2.9. Quantitative Real-Time PCR

Total RNA was isolated from liver and colon tissues by using Tri-Reagent (Sigma-Aldrich, Rehovot, Israel), according to the manufacturer’s protocol. Complementary DNA was prepared with the High-Capacity cDNA Reverse Transcription Kit (Quanta BioSciences, Gaithersburg, MD, USA). Real-time polymerase chain reaction (PCR) was performed using the 7300 Real-Time PCR System version 1.4 (Applied Biosystems, Foster City, CA, USA), with specific primers as follows: iNOS—inducible nitric oxide synthase; SAA1—Serum Amyloid A1; Fatty acid synthase (Fasn); Peroxisome proliferator-activated receptor alpha (PPARα); Sterol regulatory element-binding protein 1c (SREBP-1c); CPT-1—Carnitine palmitoyl transferase I; and CD36—cluster of differentiation 36. Quantitative changes in gene expression were determined by normalizing to 18S. The primer sequences are listed in Table 2.

### 2.10. Statistical Analysis

Results are presented as mean ± SEM. Data were analyzed by the JMP 14 Pro software suites (SAS Institute, Cary, NC, USA). Comparisons between groups were made by one-way analysis of variance (ANOVA) followed by a Tukey–Kramer test or by unpaired two-tailed Student’s *t*-test. Statistical significance was defined at *p* < 0.05.

## 3. Results

### 3.1. C. iphionoides Supplementation Reduced Body and Tissue Weight

The changes in BW, daily food intake, and adipose and liver tissue weight in each group are summarized in Table 3. Although the lowest final BW was found in ND-fed mice, both *C. iphionoides* concentrations successfully alleviated weight gain rates during the consumption of a HFD despite comparable food intake in those groups. A similar pattern of reduced liver weight in the groups that were supplemented with *C. iphionoides* compared with the HFD group was found while the weight of adipose tissue was lower only in the group that was supplemented with the higher concentrations of *C. iphionoides*.

### 3.2. C. iphionoides Supplementation Improved Liver Enzymes and Serum Lipid Profile

**Figure 1:**
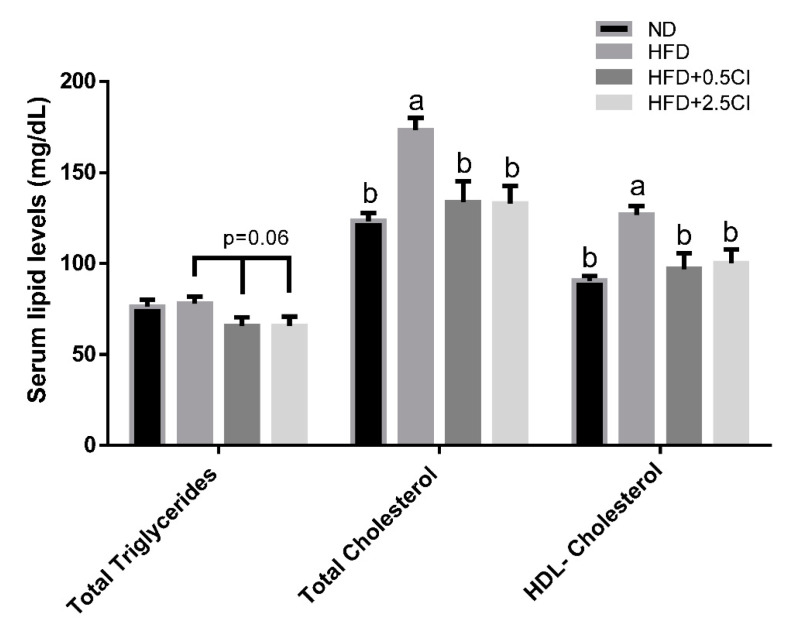
Effects of *Chiliadenus iphionoides* supplementation on serum lipid profile. Male C57BL/6J mice were fed with normal diet (ND), high-fat diet (HFD), high-fat diet + 0.5 mg/kg *Chiliadenus iphionoides* (HFD + 0.5CI), high-fat diet + 2.5 mg/kg *Chiliadenus iphionoides* (HFD + 2.5CI) for fifteen weeks. Values are expressed as mean ± SEM (*n* = 5). Different letters denote significant difference (*p* < 0.05, Tukey’s HSD).

**Figure 2:**
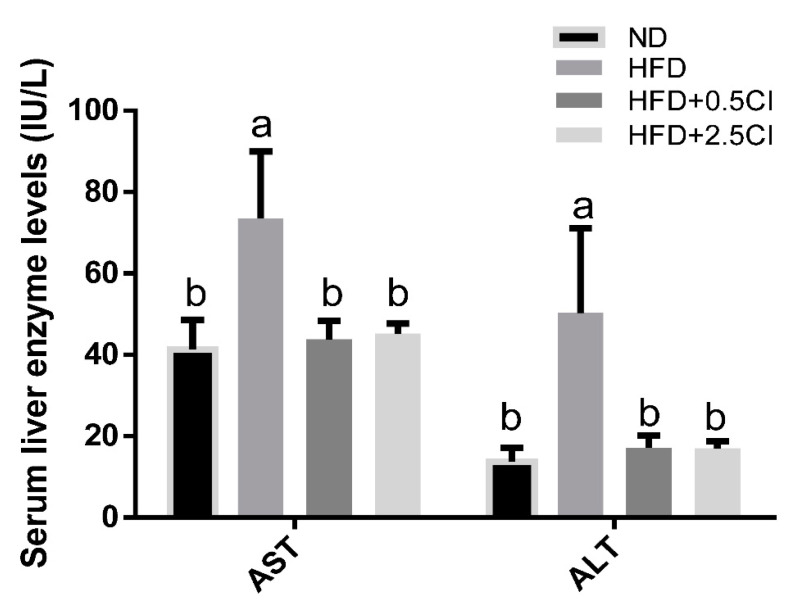
Effects of *Chiliadenus iphionoides* supplementation on serum liver enzymes. Male C57BL/6J mice were fed with normal diet (ND), high-fat diet (HFD), high-fat diet + 0.5 mg/kg *Chiliadenus iphionoides* (HFD + 0.5CI), high-fat diet + 2.5 mg/kg *Chiliadenus iphionoides* (HFD + 2.5CI) for fifteen weeks. Values are expressed as mean ± SEM (*n* = 6). Different letters denote significant difference (*p* < 0.05, Tukey’s HSD). AST—aspartate aminotransferase, ALT—alanine aminotransferase.

Total serum cholesterol and HDL levels were significantly elevated in the HFD group compared to all other groups (Figure 1). Serum triglycerides levels did not statistically differ in all groups, though a trend of reduction was noticed in the groups supplemented with *C. iphionoides* (*p* = 0.06). Liver injury due to HFD consumption was mitigated by *C. iphionoides* addition, as inferred by lower serum liver enzymes of AST and ALT (Figure 2).

### 3.3. Chiliadenus iphionoides Did Not Affect Glucose Tolerance

**Figure 3:**
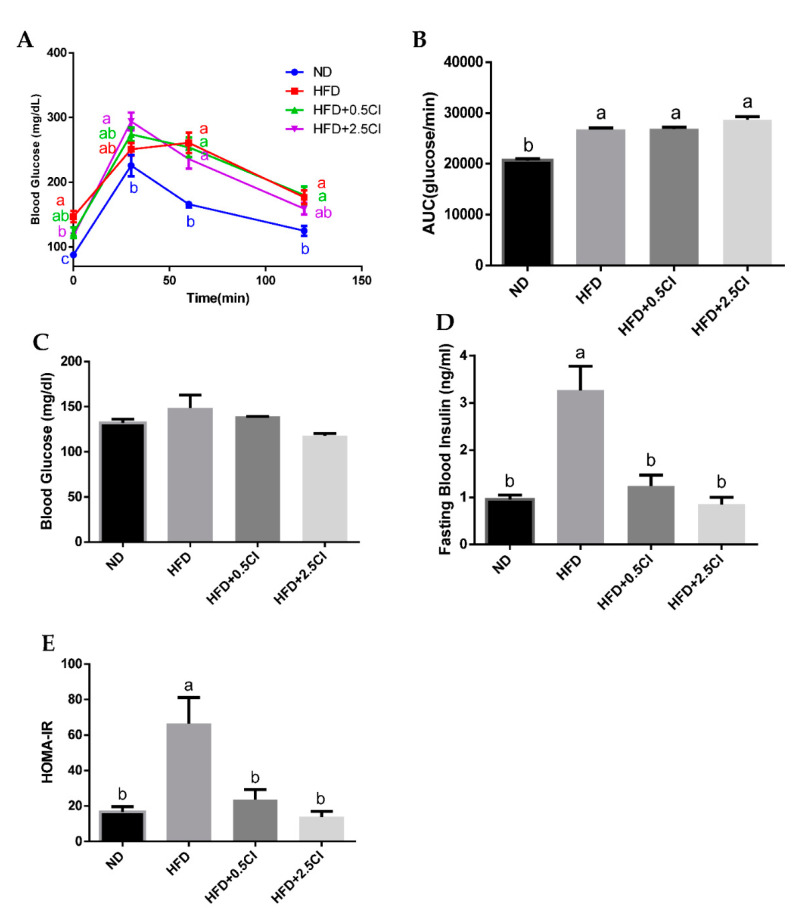
Effects of *Chiliadenus iphionoides* supplementation on glucose homeostasis. Male C57BL/6J mice were fed with normal diet (ND), high-fat diet (HFD), high-fat diet + 0.5 mg/kg *Chiliadenus iphionoides* (HFD + 0.5CI), high-fat diet + 2.5 mg/kg *Chiliadenus iphionoides* (HFD + 2.5CI) for fifteen weeks. (**A**) An oral glucose tolerance test for 120 min was performed at week 14; (**B**) Glucose tolerance test measured as the area under the curve (AUC); (**C**) Mean fasting blood glucose concentration at sacrifice; (**D**) Insulin serum levels at sacrifice; (**E**) Homeostatic model assessment of insulin resistance index (HOMA-IR). Values are expressed as mean ± SEM (ND = 9, HFD groups *n* = 10). Different letters denote significant difference (*p* < 0.05, Tukey’s HSD).

OGTT at the 13th week of the feeding experiment showed no marked effect of *C. iphionoides* addition on glucose tolerance and overall glycemic response (Figure 3A,B). However, while fasting blood glucose levels at the end of the experiment were identical between all groups (Figure 3C), plasma insulin concentrations at this time point were substantially elevated merely in the HFD group, whereas levels matched to those of the control were observed when the HFD was supplemented with *C. iphionoides* (Figure 3D). In light of the profound changes in insulin levels, the HOMA-IR index for insulin resistance was also increased only in HFD-fed mice, while it remained unchanged when this diet also contained *C. iphionoides* (Figure 3E).

### 3.4. Chiliadenus iphionoides Consumption Alleviated Liver Lipid Steatosis

**Figure 4:**
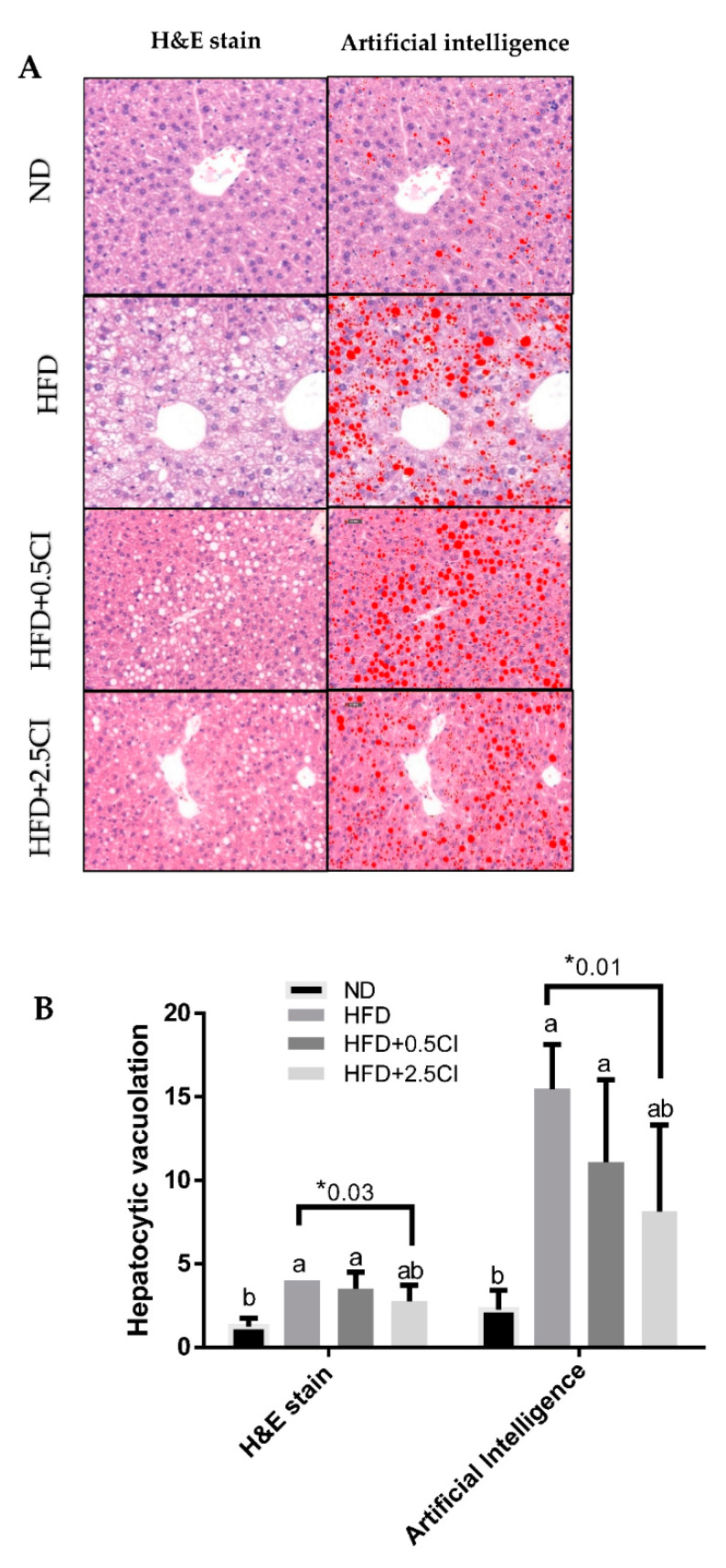
Effects of *Chiliadenus iphionoides* supplementation on histology section of the perilobular region of the liver. Male C57BL/6J mice were fed with normal diet (ND), high-fat diet (HFD), high-fat diet + 0.5 mg/kg *Chiliadenus iphionoides* (HFD + 0.5CI), high-fat diet + 2.5 mg/kg *Chiliadenus iphionoides* (HFD + 2.5CI) for fifteen weeks. (**A**) Histological evaluation of the liver using H&E stain versus artificial intelligence; (**B**) Hepatocytic vacuolation evaluated by semi-quantitive grade presented as H&E stain or by % of fatty vacuoles in hepatocytes presented by artificial intelligence. Values are expressed as mean ± SEM (*n* = 4). Different letters denote significant difference (*p* < 0.05, Tukey’s HSD) asterisk denotes significant difference (*p* < 0.05, Student’s *t*-test).

The effect of *C. iphionoides* on hepatic lipid accumulation was examined by histological semi-quantitative evaluation (H&E stain) and artificial intelligence (Figure 4A). Steatosis grade, measured using H&E stain, was greater in both HFD and HFD + 0.5CI groups compared with the control (ND). However, in the group that was supplemented with the higher concentration of *C. iphionoides* steatosis did not vary significantly from that of the control. Additional comparisons between this group and the HFD group revealed a mildly lower-grade score in the former (Figure 4B). The correlation between the semi-quantitative and AI assessment methods was estimated to be very good (r = 0.87, *p* < 0.001) [14], suggesting it is a useful tool for validating the results obtained by H&E staining. Consistent with previous results, the AI evaluation of liver steatosis revealed a comparable pattern as H&E staining, thus reinforcing these findings.

### 3.5. Chiliadenus iphionoides Impact on Genes Related to Lipid and Carbohydrate Metabolism and Inflammatory Markers

**Figure 5:**
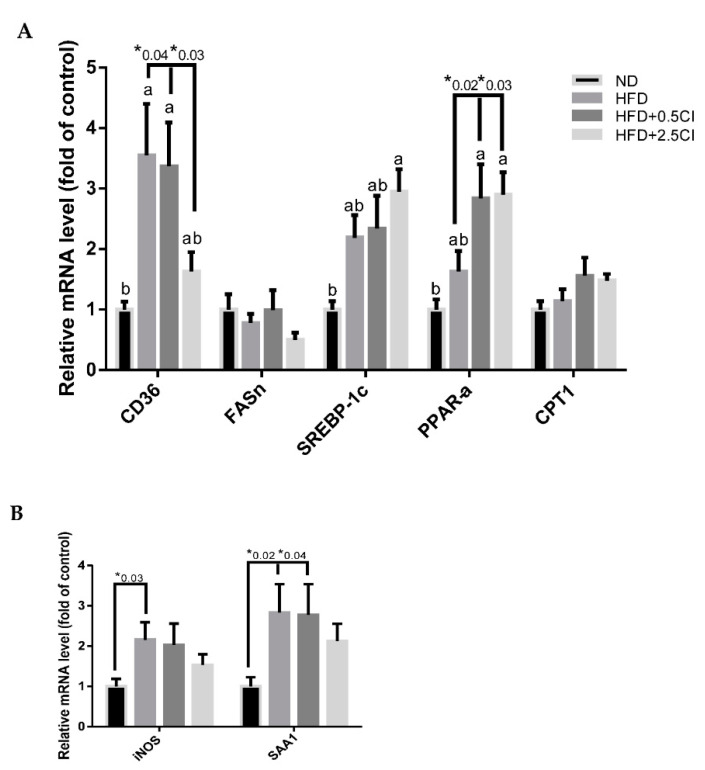
Effect of *Chiliadenus iphionoides* supplementation on genes related to liver carbohydrate and lipid metabolism. Male C57BL/6J mice were fed with normal diet (ND), high-fat diet (HFD), high-fat diet + 0.5 mg/kg *Chiliadenus iphionoides* (HFD + 0.5CI), high-fat diet + 2.5 mg/kg *Chiliadenus iphionoides* (HFD + 2.5CI) for fifteen weeks. (**A**) Gene expression of CD36, Fasn, SREBP-1c, PPAR-α, and CPT1 levels; (**B**) Gene expression of iNOS and SAA1 levels were measured. Values are expressed as mean ±SEM (ND, HFD, HFD + 0.5CI *n* = 10, HFD + 2.5CI *n* = 9). Different letters denote significant difference (*p* < 0.05, Tukey’s HSD) asterisk denotes significant difference (*p* < 0.05, Student’s *t*-test).

mRNA levels of several genes were analyzed to attempt to determine the mechanisms underlying the observed results. Expression of the fatty acids transporter CD36 increased in HFD and HFD + 0.5CI compared to the ND group, though this elevation was significantly more restricted in the latter group (Figure 5A). The conjecture that *C. iphionoides* can negatively regulate lipid uptake in the liver under obesogenic conditions was verified by the lack of divergence in CD36 expression between the group that was supplemented with the high *C. iphionoides* concentration and the control. The expression of SREBP-1c tended to increase in all HFD-fed groups, but reach significance merely in the HFD + 2.5CI group compared to ND group (Figure 5A). *C. iphionoides*-treated mice exhibited excessive mRNA levels of PPAR-α expression compared to both ND and HFD groups (Figure 5A). No significant differences were noticed between all experimental groups in FASn and CPT-1 expression (Figure 5A). The genes involved in the inflammatory response, iNOS and SAA1, similarly displayed superiority for elevated *C. iphionoides* intake during the consumption of a HFD, with both genes being upregulated by HFD but to markedly lower and insignificant levels when *C. iphionoides* was added (Figure 5B).

### 3.6. Chiliadenus iphionoides Supplementation Did Not Affect Protein Expression in Lipid and Carbohydrate Metabolism

**Figure 6:**
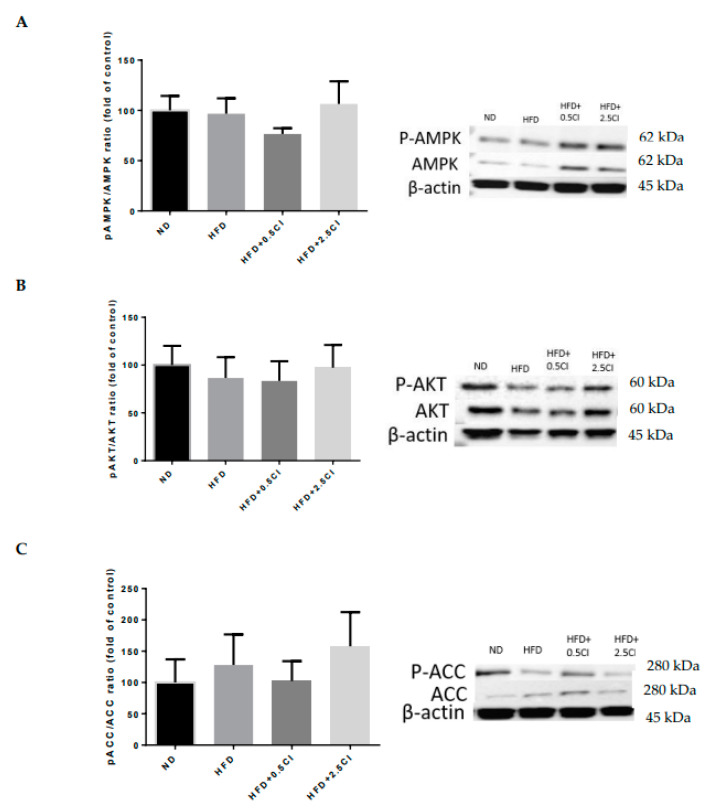
Effect of *Chiliadenus iphionoides* supplementation on proteins related to liver carbohydrate and lipid metabolism. Male C57BL/6J mice were fed with normal diet (ND), high-fat diet (HFD), high-fat diet + 0.5 mg/kg *Chiliadenus iphionoides* (HFD + 0.5CI), high-fat diet + 2.5 mg/kg *Chiliadenus iphionoides* (HFD + 2.5CI) for fifteen weeks. (**A**) Protein expression of pAMPK/AMPK ratio; (**B**) Protein expression of pAKT/AKT ratio; (**C**) Protein expression of pACC/ACC ratio. Values are expressed as mean ±SEM.

The activation or inhibition of the metabolic crossroads AMPK, AKT, and ACC, respectively, evaluated by their protein phosphorylation ratio was not statistically affected by the diets used (Figure 6A–C).

## 4. Discussion

This work is the first to describe the therapeutic potential concealed in the incorporation of *C. iphionoides* in the diet for the mitigation of liver perturbations resulting from unhealthy eating habits similar to the HFD studied. This work further supports earlier studies in favor of the utilization of plant-based treatments together with more conventional ones for metabolic disorders.

The HFD led to increased BW and hypertrophy of white adipocytes, as well as liver ectopic fat accumulation. Although the analysis conducted did not specifically address the type of adipose tissue enlargement (hypertrophy vs. hyperplastic), the overall increase in adipose tissue was significantly mitigated by *C. iphionoides* addition. Using liver weight as a measure of ectopic fat deposition, *C. iphionoides* almost completed prevented the HFD induced increase, even at a low concentration. These observations correspond with putative liver damage reflected by blood liver enzymes levels. Indeed, while liver integrity was compromised by a HFD, no such damage was found when the diet was supplemented with *C. iphionoides*. Abu-zaiton et al. [12] have found that *C. iphionoides* essential oil given orally to streptozotocin-induced diabetic rats significantly lowered blood AST levels, although ALT levels remained unaffected.

Essentially, these beneficial effects of *C. iphionoides* were conferred without any noticeable reduction in food intake, implying no diet or reduced energy intake mediated these effects. These compelling findings are of great interest considering the great difficulty many individuals experience in persisting with low-calorie diets, despite it currently being the main tool in the treatment of NAFLD.

In contrast with earlier studies [10,11,17], the assessment of glucose tolerance and overall glycemic response by OGTT did not reveal any advantageous effects for *C. iphionoides*. Indeed, blood glucose levels at almost all time points and the measured AUC exhibited similar values as those of the HFD control group. Nevertheless, a substantial and favorable effect attributed to *C. iphionoides* was obtained from the analysis of blood insulin concentrations. The addition of *C. iphionoides* profoundly hindered the observed increment in blood insulin caused by the HFD. Elevation in plasma insulin concentrations and disruptions in insulin signaling are often the repercussions of obesity and insulin resistance and constitute hallmarks of these conditions which are risk factors of NAFLD [18,19]. These findings, along with the much lower calculated HOMA-IR index clearly indicate enhanced insulin sensitivity in the groups fed *C. iphionoides*. Although it is very tempting to infer *C. iphionoides* mitigates insulin resistance caused by HFD, this is assumption is not sufficiently substantiated. The insulin-sensitivity-promoting effects ascribed to *C. iphionoides* are supported by the results obtained after fasting. However, these effects were not supported by the glucose tolerance test in the post-prandial state. Not all studies advocate that HOMA-IR is a universal measure for estimating insulin sensitivity, given the absence of constant cohesiveness between HOMA-IR and insulin-regulated postprandial glucose levels [20]. This raises the need to distinguish between conditions of overall insulin resistance with those involving only site-specific insulin resistance, i.e., central (hepatic) vs. peripheral (muscle and/or adipose tissue). It is reasonable, though unfounded [20], to surmise the present discrepancy of adequate HOMA-IR with HFD group-matched abnormal OGTT discovered in *C. iphionoides* groups may indicate this plant relieved HFD-induced central but not peripheral insulin resistance in those animals. Of course, such an inference demands more precise, comprehensive studies. It is worth mentioning that basal AKT phosphorylation ratio was not dramatically influenced by *C. iphionoides*. Therefore, one might believe this contradicts the proposed assumption. Nevertheless, it must be taken into consideration that AKT phosphorylation at Ser473 can occur in insulin-independent mechanisms under several conditions, including those that are inflammation-related [21,22,23,24,25]. As such, the usage of this parameter as a surrogate of insulin signaling in the present work is highly questionable.

Changes in lipid metabolism can be reflected by blood lipids profile as well as by key intracellular players in these pathways. These fluctuations may proceed or precede liver and/or insulin signaling alterations and thus can be referred to as the cause or the consequence of such disturbances. *C. iphionoides* addition to an HFD results in a tendency towards reduced (*p* = 0.06) TG levels and significant restoration of total cholesterol levels in the plasma. Unexpectedly, in the HFD group, HDL levels exceeded those of other groups. This finding is in accordance with previous works conducted in mice with the same genetic background in which HFD-fed mice displayed conspicuously higher total cholesterol levels, as well as greater HDL-C levels [26]. Currently, there is increasing argument against the use of total HDL as a measure for its functionality, given the piling evidence against its accuracy [27].

In relation to intracellular lipids pathways, significant differences were found in the expression of CD36 and PPARα, which participate in cellular lipid uptake [28] and upregulating β-oxidation [29], respectively. CD36 was decreased while PPARα was enhanced in the group supplemented with the higher concentration of *C. iphionoides*. These findings stand in line with those of lipids accumulation, in which an obvious propensity was noticed by this dietary manipulation. No change was found in the activity of the master metabolic regulator AMPK nor its affector ACC. It is possible that a longer diet period and/or higher extraction concentration would have led to more dramatic results.

The induction of an inflammatory state in the liver constitutes a more aggressive state of liver pathology compared with simple steatosis. In the present model, liver steatosis generated by HFD regime was accompanied by a mild induction of inflammation. The results demonstrated elevated expression levels of iNOS and SAA1; the former is a well-known enzyme implicated in the stimulation of inflammation [30], and the latter is an inducible acute phase factor that is a part of the response to injury and inflammation [31]. Mice in the high-*C. iphionoides* group appeared to further benefit from an ameliorated inflammatory state, as they exhibited reduced expression levels of both of these genes. Previously, the anti-inflammatory activity of *Varthemia iphionoides* methanolic extracts was demonstrated in cancer cells [9]. In another study, the radical-scavenging activity of ethanol and water extracts of aerial parts of *V. iphionoides* was also elucidated [32]. However, this is the first work that illustrates the anti-inflammatory effect of *C. iphionoides* in vivo.

Interestingly, other bioactive plants were documented to produce similar effects including a reduction in liver weight, liver steatosis, and inflammatory-related parameters [33]. In these studies, hepatic expression of microRNA was also downregulated, and it would be of interest in the future to evaluate the effects of *C. iphionoides* on microRNA expression.

## 5. Conclusions

In summary, the present study revealed the beneficial effects of *C. iphionoides* in a model of diet-induced obesity and NAFLD. *C. iphionoides* beneficially ameliorated increases in body weight and liver fat accumulation as well as improved blood lipid profile and liver enzymes levels. In addition to these findings, the incorporation of this plant as part of an HFD led to a concomitant improvement in fasting insulin resistance and other metabolic factors that facilitate reduction in hepatic steatosis. Based on these findings, *C. iphionoides* may be useful for preventing or retarding the development of NAFLD and metabolic complications. This study enhances awareness regarding the importance of incorporating medicinal plants in the treatment of chronic diseases such as NAFLD. Further studies are required to elucidate the effects of *C. iphionoides* in other peripheral tissues, as well as under conditions of preestablished liver steatosis and the underlying mechanisms that mediate these effects.

## Figures and Tables

**Table 1:** Compositions of animal diets.

	Normal Diet (ND)		High-Fat Diet (HFD)	
Ingredients	gr	Kcal	gr	Kcal
Casein	210	840	265	1060
L- methionine	3	12	4	16
Cornstarch	500	2000	0	0
Dextrose	100	400	160	640
Sucrose	39.15	156.6	90	360
Lard	20	180	310	2790
Soybean oil	20	180	30	270
Anhydrous milkfat	20	180	0	0
Cellulose	35	0	65.5	0
Mineral mix AIN-93G-MX (94046)	35	0	51.4	0
Vitamin mix AIN-93-VX (94047).	15	0	21	0
Choline chloride	2.75	0	3	0
BHT	0.014	0	0.014	0
Total energy (Kacl)	1000	3769	1000	5136
Fat (%)	9		60	
Protein (%)	22		21	
Carbohydrate (%)	65		19	

BHT, butylated hydroxytoluene; gr, gram.

**Table 2:** Sequences of the primers used for quantitative real-time PCR.

Name	Reverse	Forward
18s	5′-CCTCAGTTCCGAAAACCAAC-3′	5’-ACCGCAGCTAGGAATAATGG-3’
iNOS	5′-TCTCTGCTCTCAGCTCCAAG-3′	5′-AGCTCCCTCCTTCTCCTTCT-3′
SAA-1	5′-GGTCAGCAATGGTGTCCTCA-3′	5′-GATGAAGCTACTCACCAGCCT-3′
PPARα	5′-CTGCGCATGCTCCGTG-3′	5′-CTTCCCAAAGCTCCTTCAAAAA- 3′
CD36	5′-AAAGGCATTGGCTGGAAGAA-3′	5′-TCCTCTGACATTTGCAGGTCTATC-3′
Fasn	5′-GGTCGTTTCTCCATTAAATTCTCAT-3′	5′-CTAGAAACTTTCCCAGAAATCTTCC-3′
Srebp-1c	5′-TAGATGGTGGCTGCTGAGTG-3′	5′-GATCAAAGAGGAGCCAGTGC-3′
CPT-1	5′-CAGCGAGTAGCGCATAGTCA-3′	5′-TGAGTGGCGTCCTCTTTGG-3′

iNOS—inducible nitric oxide synthase; SAA1—Serum Amyloid A1; Fatty acid synthase (Fasn); Peroxisome proliferator-activated receptor alpha (PPARα); Sterol regulatory element-binding protein 1c (SREBP-1c); CPT-1—Carnitine palmitoyl transferase I; CD36—cluster of differentiation 36.

**Table 3:** Effects on food intake, body, and tissue weight.

	Group			
	ND (n = 10)	HFD (n = 10)	HFD + 0.5CI (n = 10)	HFD + 2.5CI (n = 10)
Initial body weight (g)	22.06 ± 0.20 ^a^	21.76 ± 0.34 ^a^	21.17 ± 0.31 ^a^	21.39 ± 0.23 ^a^
Final body weight (g)	27.19 ± 0.41 ^c^	40.63 ± 1.23 ^a^	35.65 ± 1.06 ^b^	32.99 ± 1.13 ^b^
Food intake (g/day)	3.27 ± 0.48 ^a^	2.87 ± 0.45 ^b^	2.64 ± 0.34 ^b^	2.75 ± 0.31 ^b^
Adipose tissue weight (g)	0.60 ± 0.06 ^b^	1.8 ± 0.12 ^a^	1.59 ± 0.12 ^ab^	1.17 ± 0.16 ^b^
Liver tissue weight (g)	0.96 ± 0.03 ^b^	1.32 ± 0.07 ^a^	1.10 ± 0.05 ^b^	1.02 ± 0.05 ^b^

Effects of *Chiliadenus iphionoides* supplementation on weight gain, food intake, and tissues weight. Male C57BL/6J mice were fed with normal diet (ND), high-fat diet (HFD), high-fat diet + 0.5 mg/kg *Chiliadenus iphionoides* (HFD + 0.5CI), high-fat diet + 2.5 mg/kg *Chiliadenus iphionoides* (HFD + 2.5CI) for fifteen weeks. Values are expressed as mean ± SEM. Different letters denote significant difference (*p* < 0.05, Tukey’s HSD).
